# Cortical bone thickness on preoperative CT scans as predictor of bone quality in distal femur fractures: a retrospective study in Caucasians

**DOI:** 10.1007/s00402-023-05131-2

**Published:** 2023-12-04

**Authors:** Calvin M. Huppke, Hendrik Fahlbusch, Karl-Heinz Frosch, Matthias Krause, Fidelius von Rehlingen-Prinz

**Affiliations:** 1 Department of Trauma and Orthopaedic Surgery, University Medical Center Hamburg-Eppendorf, Martinistraße 52, 20251 Hamburg, Germany; 2 Department of Trauma Surgery, Orthopaedics and Sports Traumatology, BG Hospital Hamburg, Bergedorfer Str. 10, 21033 Hamburg, Germany; 3 Klinik und Poliklinik für Unfallchirurgie und Orthopädie, Universitätsklinikum Hamburg-Eppendorf, Martinistraße 52, 20246 Hamburg, Deutschland

**Keywords:** Distal femur fracture, Cortical bone thickness, Osteoporosis, CT scan, Knee joint

## Abstract

**Aim:**

Distal femur fractures (DFF) are rare, but associated with high complication rates and mortality, particularly in patients with osteoporosis. To improve preoperative assessment, we analyzed if cortical bone thickness on CT and AP radiographs is associated with clinical parameters of bone quality.

**Methods:**

Retrospective single-center study of adult patients presenting at a level-one trauma center, with a DFF between 2011 and 2020. Clinical parameters for bone quality, such as age, sex, body mass index (BMI), energy impact level of trauma, and known history of osteoporosis, were assessed. Mean cortical bone thickness (CBTavg) on AP radiograph was determined using a previously published method. Cortical thickness on CT scan was measured at 8 and 14 cm proximal to the articular surface of the lateral condyle.

**Results:**

71 patients (46 females) between 20 and 100 years were included in the study. Cortical thickness determined by CT correlated significantly with CBTavg measurements on AP radiograph (Spearman *r* = 0.62 to 0.80; *p* < 0.001). Cortical thickness was inversely correlated with age (Spearman *r* = − 0.341 to − 0.466; *p* < 0.001) and significantly associated with trauma impact level and history of osteoporosis (*p* =  < 0.001). The CT-based values showed a stronger correlation with the clinical parameters than those determined by AP X-ray.

**Conclusion:**

Our results showed that cortical thickness of the distal femur correlates with clinical parameters of bone quality and is therefore an excellent tool for assessing what surgical care should be provided. Interestingly, our findings indicate that cortical thickness on CT is more strongly correlated with clinical data than AP radiograph measurements.

**Supplementary Information:**

The online version contains supplementary material available at 10.1007/s00402-023-05131-2.

## Introduction

Distal femur fractures (DFF) are rare with a prevalence of 0.4% among all fractures, but account for 3–6% of all femoral fractures [1]. Epidemiological studies have shown that sex, age, and cause of fracture follow a bimodal distribution pattern [2]. Younger patients are predominantly male and present with high-impact trauma, such as motor vehicle accidents [2]. In contrast, the second and larger demographic group comprises elderly women with osteoporosis in whom low-impact trauma, such as a fall from standing height, is the main cause of injury [3, 4].

Due to the rarity and frequent intraarticular involvement in up to 53% of cases, distal femur fractures are still considered a serious challenge [5]. Complications with malunion, nonunion, implant failure, and wound infection have been reported in 19% to 32% of cases [6, 7]. Consequently, many different surgical techniques have been developed including condylar buttress plates, dynamic condylar screws, angle blade plates, locking compression plates, dual plating, as well as antero- and retrograde nailing [8–10].

Due to the aging population, an increasing technical challenge has become the osteoporotic fracture. Thin cortices and loss of bone stock can be the cause of inadequate fixation, implant failure, and delayed rehabilitation [11–13]. Consequently, modern studies suggest the necessity for more rigid, reliable, and stable fixation methods, such as dual plating, to manage these types of fractures [10].

Since it is estimated that over half of all distal femur fractures are associated with osteoporosis, consideration of bone mineral density (BMD) is essential in operative planning for the elderly to reduce hospital stays, accelerate remobilization and prevent complications [14].

The current clinical gold standard to determine osteoporosis-related bone fragility is dual-energy X-ray absorptiometry (DXA) measurement of BMD [15]. However, DXA is not routinely used as an investigation in asymptomatic patients, which is why an osteoporotic fracture can be the first sign of degenerative bone loss [16, 17].

Recent studies of cortical thickness on anteroposterior (AP) radiographs have demonstrated a correlation with BMD measured by DXA [15, 18, 19]. He et al. [18] were the first to develop a measurement technique to assess mean cortical bone thickness (CBTavg) of the distal femur on AP radiographs. In a prospective study on 361 healthy adults, they demonstrated a strong correlation between CBTavg and BMD (*r* = 0.664, *p* < 0.01) [18].

In our study, we aimed to expand on the results of He et al. by examining cortical thickness in a large cohort of patients with DFF and correlate the data with clinical parameters of bone quality. Moreover, as the preoperative work-up in the majority of patients with distal femur fractures includes a CT scan to assess intraarticular involvement, our second objective was to develop a method to determine cortical thickness in these scans [20, 21].

## Methods

### Patients

This retrospective single-center study was performed in a level-one trauma center. Inclusion criteria were age > 18 years, a documented distal femur fracture between the years 2011 and 2020, a preoperative axial bone window CT scan (window thickness: 2500, level: 500) and an AP X-ray radiograph. Patients with primary or metastatic bone tumors or proliferative bone disorders were excluded. Patient-specific data included age, sex, BMI, energy impact level of the fracture, and a documented diagnosis of osteoporosis in the patient’s history (OST history). Fractures were classified based on the AO/OTA system and all subgroups of fractures were included (OTA / AO 33-A, 33-B, 33-C; 32-A, 32-B, 32-C) [22]. The cases were divided into low- and high-impact trauma groups. Low-impact trauma was defined as a spontaneous fracture or fall from standing height; high-impact trauma was defined as a vehicle accident or fall from a height greater than five meters [2]. A positive OST history was noted if a diagnosis of osteoporosis was listed as a known comorbidity in the patient's medical history at the time of the accident.

### Measurement of cortical thickness

The CBTavg on AP radiographs was calculated using the method described by He et al. [18] Hereby, the difference in distance between the endocortical and outer cortical border was measured at two levels and the mean of the two measurements then calculated.

Axial CT scans were analyzed to measure cortical bone thickness by applying the computerized on-screen measurement tool of the Picture Archiving and Communication System (PACS). Cortical thickness was determined at two different levels of the distal femur, defined as the supracondylar (SC) and distal shaft (DS) level. The SC measurement was chosen for its close proximity to the fracture location. The DS position was selected as cortical thickness increases toward the midshaft region of the femur thereby improving measurement reliability.

**Fig. 1:**
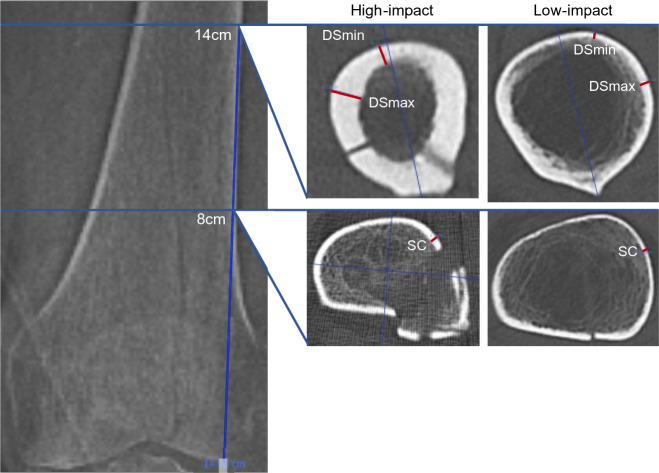
Schematic diagram of a preoperative axial bone window CT scan (window thickness: 2500, level: 500). Blue lines depicting SC measurement at 8 cm and the DSmax and DSmin measurement at 14 cm proximal to the articular surface of the knee joint. The axial view shows points of measurements in red, demonstrating variances of cortical thickness in cases of high- and low-impact trauma

SC cortical thickness was determined at 8 cm proximal to the articular surface of the lateral condyle as this point, according to AO OTA definition, represents the most proximal region of what is still considered part of the distal femur [22, 23]. Although cortical thickness at this level did not show any variation between quadrants, to standardize the measurement, the cortical thickness was measured in the anterolateral quadrant as shown in supplementary (Fig. 1).

DS cortical thickness was measured at 14 cm proximal to the articular surface of the lateral condyle as it represents the most proximal part of the femur included in the standard CT imaging. In contrast to the SC measurement, cortical thickness varies significantly at the 14 cm plane. Therefore, both the minimal (DSmin) and the maximal (DSmax) cortical thicknesses were determined. The region of the linea aspera was excluded. To standardize measurements, the femur was divided by a line passing through the shaft center and the linea aspera, and the DSmax and the DSmin were measured in the lateral half of the shaft (Fig. 1).

To determine the axial plane for the cortical measurements, the associated topography was aligned with the axial scan window on-screen. The correct axial plane was determined by spanning the measurement tool parallel to the lateral border of the femoral shaft from the articular surface of the lateral condyle proximally to 8 cm and 14 cm, respectively (Fig. 1). In case of shaft dislocation, two separate measurement lines were fitted to find the correct axial plane as shown in Fig. 1. To measure the cortical thickness in mm, the endocortical border had to be distinguished from the lower density transitional zone of the trabecular bone [24].

### Statistical analyses

The data were processed using IBM SPSS Statistics software for Windows, version 27 [25]. Descriptive statistics report counts categorical variables, otherwise means and standard deviation as well as 95% confidence intervals (Table 1). Prior to the parametric test for bivariate analyses, an F-test was applied to assess differences in variance between two groups. In accordance with the results, the Welch´s t-test was chosen to determine significant differences between these groups (Table 2). The Spearman correlation test was used to assess the relationship between cortical thickness measurements on CT (SC, DSmin, and DSmax) and age and CBTavg on AP radiograph (Table 3). Additionally, a multiple linear regression model was fitted to assess the ability of the five clinical factors (sex, age, BMI, impact level of trauma, and OST history) to predict cortical thickness on CT and AP radiograph (Table 4). No adjustment for potential confounders was made. The P value to indicate significance was set at < 0.001.

**Table 1:** Patient characteristics n = 71

	Sex		Age (yrs)	BMI (kg/m2)	OST history		Impact type	
	Male *n*	Female *n*	Mean (SD)	Mean (SD)	No diagnose *n*	Diagnosed n	High-impact *n*	Low-impact *n*
Sex								
Male	25		53 (17)	27.8 (3.9)	23	2	20	5
Female		46	71 (18)	26.5 (10.8)	27	19	8	38
OST history								
No diagnosis	23	27	59 (18)	28.1 (9.7)	50		27	23
Diagnosed	2	19	78 (16)	23.9 (5.7)		21	1	20
Impact type								
High-impact	20	8	49 (14)	27.7 (4.7)	27	1	28	
Low-impact	5	38	74 (16)	26.7 (11)	23	20		43
Revision surgery	6	9	62 (17)	30 (10)	3	12	6	9

*OST* osteoporosis, *BMI* body mass index

**Table 2:** Univariate analyses of cortical measurements (mm)

Variable	SC measurement (mm)			DSmin measurement (mm)			DSmax measurement (mm)			CBTavg (mm)		
	Mean (SD)	Median	p-value	Mean (SD)	Median	p-value	Mean (SD)	Median	p-value	Mean (SD)	Median	*p*-value
Sex												
Female	1.28 (0.4)	1.2	< 0.001	2.39 (1)	2.3	< 0.001	3.12 (1.3)	3	< 0.001	4.33 (1.1)	4.4	< 0.001
Male	1.74 (0.3)	1.8		3.60 (0.8)	3.7		4.73 (1)	4.7		5.31 (0.8)	5.3	
OST history												
Yes	1.16 (0.3)	1.1	< 0.001	2.03 (0.8)	2	< 0.001	2.64 (1.2)	2.4	< 0.001	3.75 (1.2)	3.8	< 0.001
No	1.57 (0.4)	1.5		3.15 (1)	3.25		4.21 (1.2)	4.35		5.06 (0.8)	5.0	
Impact level												
Low-impact	1.27 (0.4)	1.2	< 0.001	2.16 (0.8)	2.1	< 0.001	2.98 (1.2)	2.8	< 0.001	4.15 (1)	4.2	< 0.001
High-impact	1.72 (0.3)	1.75		3.84 (0.6)	4		4.91 (0.8)	4.9		5.47 (0.7)	5.3	
Sex/Impact level												
Female												
Low-impact	1.24 (0.4)	1.15	0.079	2.12 (0.8)	2	< 0.001	2.89 (1.2)	2.75	< 0.001	4.13 (1.1)	4.2	0.003
High-impact	1.49 (0.2)	1.5		3.71 (0.5)	3.6		4.71 (0.5)	4.85		5.26 (0.7)	5.1	
Male												
Low-impact	1.46 (0.2)	1.4	0.23	2.46 (0.6)	2.3	< 0.001	3.66 (0.8)	3.9	0.005	4.31 (0.2)	4.3	< 0.001
High-impact	1.81 (0.3)	1.85		3.89 (0.6)	4.05		5.00 (0.9)	4.9		5.56 (0.7)	5.4	

Significant difference (*p* < 0.001); *SD* standard deviation, *OST* osteoporosis, *BMI* body mass index, *CBTavg* mean cortical bone width, *AP* anteroposterior, *r* Spearman correlation coefficient, *SC* supracondylar measurement, *DSmin* distal shaft minimal cortical width, *DSmax* distal shaft maximum cortical width

**Table 3:** Correlation between cortical thickness on CT scan and CBTavg on AP radiograph and age

	CBTavg (mm)		Age (years)	
	*r*	*p*-value	*r*	*p*-value
SC (mm)	0.619	< 0.001	−0.341	0.004
DSmin (mm)	0.708	< 0.001	−0.466	< 0.001
DSmax (mm)	0.804	< 0.001	−0.440	< 0.001

*CBTavg* mean cortical bone width, *AP* anteroposterior, *r* Spearman correlation coefficient, *SC* supracondylar measurement, *DSmin* distal shaft minimal cortical width, *DSmax* distal shaft maximum cortical width

**Table 4:** Linear multiple regression analysis assessing clinical predictors cortical thickness on CT scan and AP radiograph in patients with distal femur fractures

	CT scan									AP radiograph		
	SC (mm)			DSmin (mm)			DSmax (mm)			CBTavg (mm)		
	*B (SE)*	*P*	*R*^*2*^	*B (SE)*	*P*	*R*^*2*^	*B (SE)*	*P*	*R*^*2*^	*B (SE)*	*P*	*R*^*2*^
Clinical Model		< 0.01	0.44		< 0.01	0.6		< 0.01	0.53		< 0.01	0.44
Sex	− 0.253 (0.1)	0.014		− 0.204 (0.226)	0.371		− 0.406 (0.314)	0.202		− 0.145 (0.264)	0.586	
Age (yrs)	0.004 (0.003)	0.166		0.004 (0.006)	0.465		0.006 (0.008)	0.425		0.004 (0.007)	0.581	
BMI (kg/m2)	0.010 (0.004)	0.030		0.017 (0.010)	0.091		0.029 (0.014)	0.044		0.024 (0.012)	0.047	
Impact level	− 0.272 (0.113)	0.019		− 1.485 (0.253)	< 0.001		− 1.49 (0.355)	< 0.001		− 0.955 (0.298)	0.002	
OST history	− 0.191 (0.100)	0.030		− 0.270 (0.226)	0.091		− 0.604 (0.301)	0.060		− 0.642 (0.264)	0.047	

*AP* anteroposterior, *SC* supracondylar cortical thickness, *DSmin* distal shaft minimum cortical thickness, *DSmax* distal shaft maximum cortical thickness, *CBTavg* mean cortical bone thickness on AP radiograph, *OST* osteoporosis, *BMI* body mass index, *B* unstandardized coefficient B, *SE* standard error

## Results

**Fig. 2:**
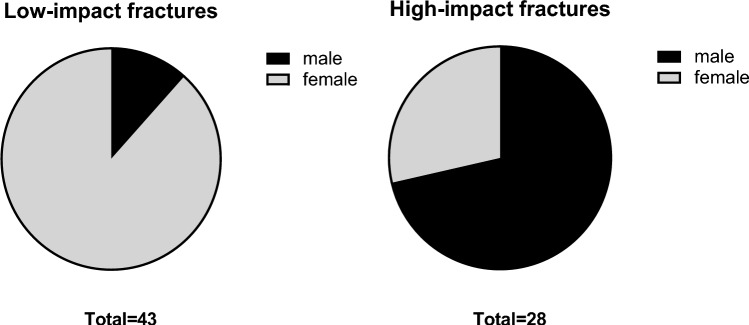
Gender distribution by high- and low-impact trauma

**Fig. 3:**
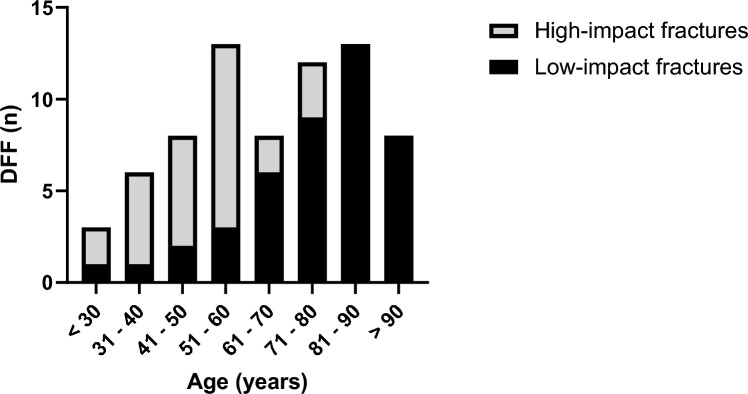
Age distribution for the total number of DFF stratified by impact type

A total of 132 patients with DFF were identified in the database, of which 77 cases met age, timeframe, and radiological criteria. Two patients were subsequently excluded due to the presence of a metastatic bone lesion or proliferative bone disorder, and an additional four patients were excluded due to insufficient clinical data. The demographic and clinical data of 71 patients (65% female, *n* = 46), aged 20–100 years, included in this study are presented in Table 1. The most common injury mechanism was low-impact trauma (61%, *n* = 43). Patients with low-impact fractures were predominantly female (88%, *n* = 38) and older than patients with high-impact trauma (mean age [SD]: 74 years [16] vs. 49 years [14]) (Figs. 2 and 3). Conversely, high-impact trauma was more common among males (71%, *n* = 20) (Fig. 2). A history of osteoporotic bone fragility was documented in 47% (*n* = 20) of the low-impact trauma patients. Among the study group, 15 patients (Table 1) required surgical revisions due to implant failure, pseudarthrosis, or infection.

**Fig. 4:**
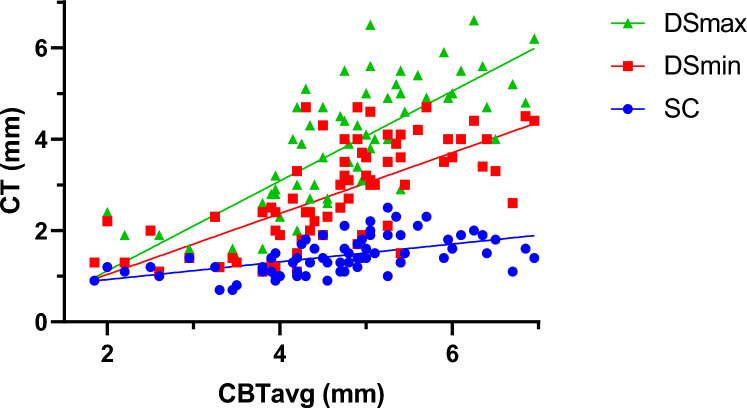
Correlation between mean cortical bone thickness (CBTavg) on AP radiograph and three measurements of cortical bone thickness (SC, DSmin, DS max) on CT scan. Showing all data points

**Fig. 5:**
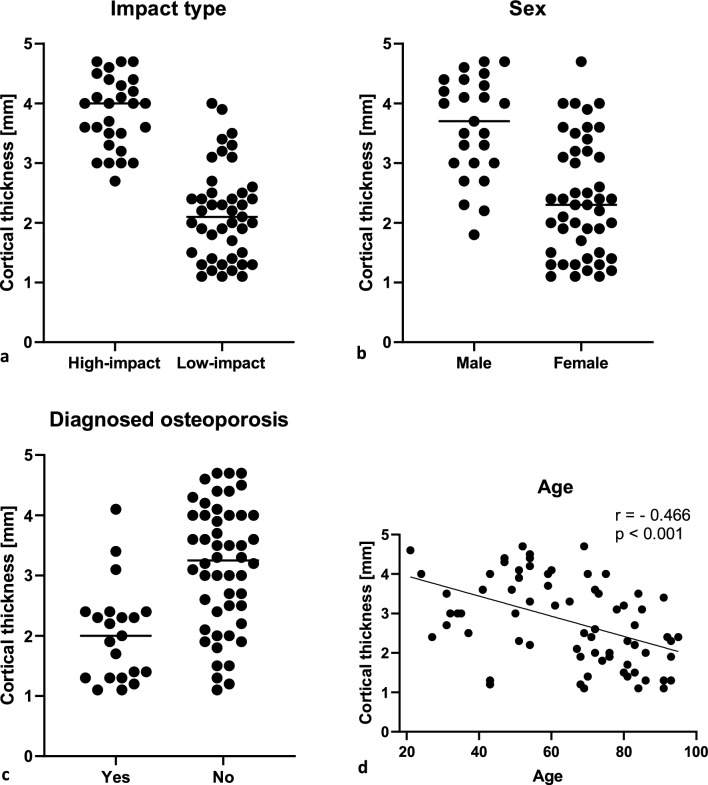
Associations between DSmin cortical thickness and clinical parameters, showing all separate data points

All three CT measurements (SC, DSmin, DSmax) correlated significantly with CBTavg measured on AP radiographs (Spearman *r* = 0.62 to 0.80; *p* < 0.001) (Fig. 4, Table 3). The strongest correlation with radiographic data was observed for the DSmax measurement (r = 0.804). Associations between cortical thickness and clinical parameters are presented in Table 2 and further illustrated in Fig. 5. Cortical thickness on CT scans was significantly lower in patients who experienced low- compared to high-impact trauma (*p* < 0.001) (Table 2, Fig. 5, supplementary Fig. 1). Age was inversely correlated with cortical thickness with the strongest correlation observed for DSmin (Spearman *r* = − 0.466, *p* < 0.001) (Table 3, Fig. 5). A significant difference in cortical thickness between high- and low-impact trauma was found in both sexes for the DSmin and the DSmax measurements, but not for the SC measurement (Table 2). Regardless of impact type, males had a higher mean cortical thickness value at all three measurement points (Table 2). Finally, patients with a documented history of osteoporotic bone fragility were found to have significantly lower cortical thickness (Table 2, Fig. 5).

A multiple linear regression model was used to assess the influence of five clinical predictors (age, sex, BMI, trauma impact level, and history of osteoporosis) on cortical thickness on CT (Table 4). The model was significant for all measurement points (F (4.66) = SC: 12.4; DSmin: 25; DSmax: 18.6; p < 0,001). The highest predictive value of the model was observed for the DSmin measurement (SC: DSmin: R^2^ = 0,603 vs. DSmax: R^2^ = 0,529 and SC: R^2^ = 0,429), explaining 60% of the variance in cortical thickness. Impact type was shown to have the greatest influence as an independent predictor for all measurements. The clinical model was also found to be predictive of CBTavg on AP radiographs. However, the predictive value (R^2^ = 0.442) was lower than that shown for DS measurements on CT.

## Discussion

The main findings of this study showed that cortical thickness, measured in preoperative CT scans of DFF patients, exhibited a significant correlation with established indicators of poor bone quality and validated X-ray measurements (*p* < 0.01) (Tables 3 and 4). Notably, CT measurements demonstrated superior accuracy when compared to conventional X-ray techniques based on clinical data. These results suggest that the presented cortical measurements provide a valuable insight into bone quality, offering guidance to surgeons in selecting the appropriate fixation methods.

Distal femur fractures continue to present a complex challenge for orthopedic surgeons, as unsatisfactory treatment outcomes, such as nonunion, implant failure, and late loss of alignment, remain common [6, 7, 26]. Particularly, an aging population has led to a rise in osteoporotic fractures, which can prove challenging as the poor bone quality may result in unstable fixation and delay rehabilitation [10–13]. This study provides a comprehensive analysis of preoperative radiological and clinical data associated with degenerative bone loss in patients with DFF. It aims to identify poor bone quality and help guide future surgical procedures. The main radiological outcome measure chosen to signify reduced bone quality was the cortical thickness in the distal femur on anteroposterior (AP) radiographs and CT (Fig. 1). The chosen method of measurement applied on AP radiographs (CBTavg) was established by He et al. [18]. In CT scans, cortical thickness was analyzed at three standardized measurement points (SC, DSmin, Dsmax; Fig. 1). First, it was assessed whether the CT measurements correlated with the established methods from the AP radiographs. Notably, a significant correlation was observed across all three measurement points (p < 0.01), with the strongest correlation seen at the point of maximal cortical thickness (DSmax) located 14 cm proximal to the articular surface of the lateral condyle (*p* < 0.01, *r* = 0.804) (Table 3). Several studies have shown that cortical thickness measured on AP radiograph of the fracture site correlates with the bone mineral density (BMD) results from DXA [16, 18, 19, 27–30]. Most of these studies have focused on the metaphyseal regions of the proximal humerus, proximal femur, femoral shaft, proximal tibia, and distal tibia, as these are common regions for osteoporotic fractures. To date, only He et al. have addressed the distal femur [18]. They found a strong correlation between cortical thickness from AP knee radiographs and femoral BMD results from DXA scans in 361 healthy adults (*r* = 0.664, *p* < 0.01) [18]. In contrast to this study, which was based on healthy adults, our study focused on the clinical application of cortical measures in a cohort of patients with DFF. In addition, we investigated whether cortical bone thickness in DFF patients correlated with clinical parameters of bone quality. We also investigated whether preoperative CT scans, which are commonly performed in most patients, provide an improved means of assessing bone quality using cortical measures. Matching previous reports, a bimodal pattern of distribution was found, with high-impact fractures seen predominantly in young males (mean age 49 years) and associated with high-velocity accidents or falls from a great height while low-impact fractures following a fall from standing height were more common in older females (mean age 74 years) (Table 1, Fig. 2) [2]. As seen in epidemiological studies, these results indicate that bone quality varies considerably among DFF patients [2–4]. Analyzing the radiological data, it was found that the cortical thickness from preoperative imaging showed a strong correlation with relevant clinical determinants of bone quality (*p* < 0.01) (Table 4). We found significantly higher mean cortical thickness in males than females and a loss of cortical thickness with increasing age (Fig. 5). In women, age has been observed to be associated with a 53–58% reduction in femoral bone mass over a lifetime, while in men, there is a decrease of about two-third of this amount [31]. Consistent with the bimodal distribution, cortical thickness was significantly lower in patients who suffered from low-impact trauma compared to high-impact trauma (*p* < 0.01) (Table 2, Fig. 5). Finally, patients with a documented diagnosis of osteoporosis were found to have significantly lower cortical thickness (*p* < 0.01). Notably, in this study, only 47% of patients presenting with low-impact trauma to the distal femur had previously been diagnosed with osteoporosis (Table 1). For the remainder, the surgeon either assumed physiologic bone structure or relied on experience, and image assessment or patient characteristics to predict likely bone quality. As our results show a strong correlation between impaired BMD and CT cortical thickness, cortical thickness may provide an additional objective clinical parameter for assessing bone quality. In our analyses, cortical thickness measured by CT scan showed higher correlation with clinical parameters than cortical thickness determined by X-ray radiograph (Table 4). The method presented is quick and easy to apply. The axial view provides a direct view of the cortical borders, allowing reliable measurement of cortical thickness. This innovative approach allows surgeons to gain information about bone quality, enabling them to select more robust and stable fixation techniques for fragile bone cases. Both, X-ray or CT-based methods, have the advantage over DXA in that they do not require any additional examinations. X-rays or CT scans are routinely performed prior to surgery, and the thickness of the cortex may be measured directly at the site of the proposed surgery, saving time, and improving the diagnostic process. In our cohort, 15 patients (21%) required revision surgery due to implant failure, pseudarthrosis, or infection, a finding consistent with recent studies (6, 7). Due to the limited sample size, this group did not have sufficient statistical power to conclude whether the chosen surgical technique, in relation to cortical thickness, exhibited an impact on the surgical outcomes. The occurrence of complications is a complex process influenced by multiple factors, including not only cortical bone quality, but also fracture morphology and various patient-specific risk factors (7). It is worth noting that within the lowest fifth percentile of DSmin values, three out of five patients in this subgroup presented with complications. Notably, all of these cases were treated using unilateral plate osteosynthesis. We postulate that for these patients, a more robust and stable fixation method, such as dual plating, may reduce the risk of complications, potentially leading to better clinical outcomes.

Limitations of this study were its retrospective nature and unavailability of DXA data. Furthermore, as the vast majority of our patients were Caucasians, our finding might not be applicable to other ethnic groups.

In conclusion, we have been able to show that the cortical thickness measured by a CT-based method has a higher correlation with clinical parameters that indicate poor bone quality than the cortical thickness measured by X-ray radiography. This study provides surgeons with a new, easy-to-use tool to assess bone quality and tailor surgical treatment accordingly.

### Supplementary Information

Below is the link to the electronic supplementary material.

Supplementary file1 (DOCX 44 KB)

## Data Availability

The data that support the findings of this study are available from the corresponding author, M. K., upon reasonable request.
