# Supplementary material for: Cortical bone thickness on preoperative CT scans as predictor of bone quality in distal femur fractures: a retrospective study in Caucasians
Source: Arch Orthop Trauma Surg. 2023 Dec 4;144(2):731–40. doi: 10.1007/s00402-023-05131-2 (PMC10822795; doi:10.1007/s00402-023-05131-2)
Supplement: Supplementary file 1 — Supplementary file1 (DOCX 44 KB) [file 402_2023_5131_MOESM1_ESM.docx]

**Fig. 1** Distribution of cortical thickness (in mm) at three measurement points categorized by impact level (N=71).

Abbreviations: HI, High-impact; LI, Low-impact; SC, supracondylar cortical thickness; DSmin, distal shaft minimum cortical thickness; DSmax, distal shaft maximum cortical thickness
